# Left ventricular perforation after transatrial delivery of a balloon-expandable valve

**DOI:** 10.1016/j.xjse.2025.100059

**Published:** 2025-07-03

**Authors:** Christine Yang, Michael Salna, Luigi Pirelli, Isaac George

**Affiliations:** Division of Cardiothoracic Surgery, Department of Surgery, Columbia University Medical Center, New York, NY

**Figure:**
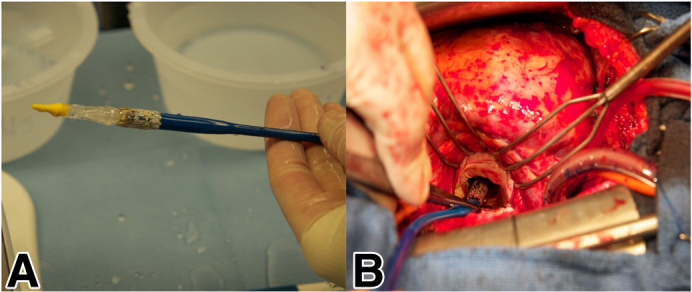
A, Sapien 3 deployment system. B, Aligned Sapien 3 valve advancement over wire.

**Central Message**

We report 3 cases of left ventricular perforation caused by the delivery system nose cone and offer suggestions on how to repair and avoid this complication.

Patients with symptomatic, severe mitral stenosis (MS) with significant mitral annular calcification (MAC) may be higher-risk candidates for traditional mitral valve replacement (MVR). Extensive calcium debridement in standard MVR can result in devastating complications, such as atrioventricular groove disruption or myocardial infarction.^1^ While surgical management of severe MS with MAC continues to evolve, novel transcatheter options are expanding access for patients previously considered inoperable.

At present, in high-risk patients for whom a transcatheter or traditional surgical option is not feasible as determined by experienced surgeons, a hybrid strategy involving cardiopulmonary bypass (CPB) and direct transatrial implantation of a balloon-expandable transcatheter aortic valve remains an attractive and viable option.

The technical steps of this “Sapien in MAC” strategy have been outlined elsewhere.^2^ As more of these procedures are performed, unexpected new complications have arisen. The Institutional Review Board or equivalent ethics committee of the Columbia University Irving Medical Center approved the study protocol and publication of data (AAK3154; approved May 14, 2025). All patients provided informed written consent for the publication of the study data (Table 1).

**Table 1:** Baseline characteristics of the 3 patients in this case series

Characteristic	Case 1	Case 2	Case 3
Sex	Female	Female	Female
Age, y	75	70	84
Diagnosis	Severe MS	Severe MS, severe TR, paroxysmal atrial fibrillation	Severe mitral regurgitation and CHF s/p MVR in 2002
Body surface area, m^2^	2.32	1.75	1.65
Preoperative echocardiography data			
LVEF, %	65	55	45
Preoperative right heart catheterization			
PCWP	7	29	8
Previous history of cardiac surgery	None	None	Tissue mitral valve replacement (#33 Hancock II placed 2 y earlier)
Intervention	Mitral valve replacement (Sapien in MAC), ligation of LAA, left atrial ablation (Box + CTI), Sentinel cerebral filter insertion, myectomy	Mitral valve replacement (Sapien in MAC), septal myomectomy, tricuspid valve replacement (29-mm EIPC) and cryo-maze procedure	Open redo mitral valve-in-valve (Sapien XT 26 mm in old Hancock II 27-mm valve stent)
Device	Sapien 3 Ultra transcatheter valve, 29 mm	Sapien 3 Ultra transcatheter valve, 29 mm	Sapien XT transcatheter valve, 26 mm
CPB time, min	3 h, 32 min + 1 h, 2 min Total: 4 h, 34 min	1 h, 5 min + 33 min Total: 1 h, 38 min	46 min + 33 min Total: 1 h, 19 min

*MS*, Mitral stenosis; *TR*, tricuspid regurgitation; *CHF*, congestive heart failure; *MVR*, mitral valve replacement; *LVEF*, left ventricular ejection fraction; *PCWP*, pulmonary capillary wedge pressure; *MAC*, mitral annular calcification; *LAA*, left atrial appendage; *CPB*, cardiopulmonary bypass.

## Case Descriptions

### Case 1

**Figure 1:**
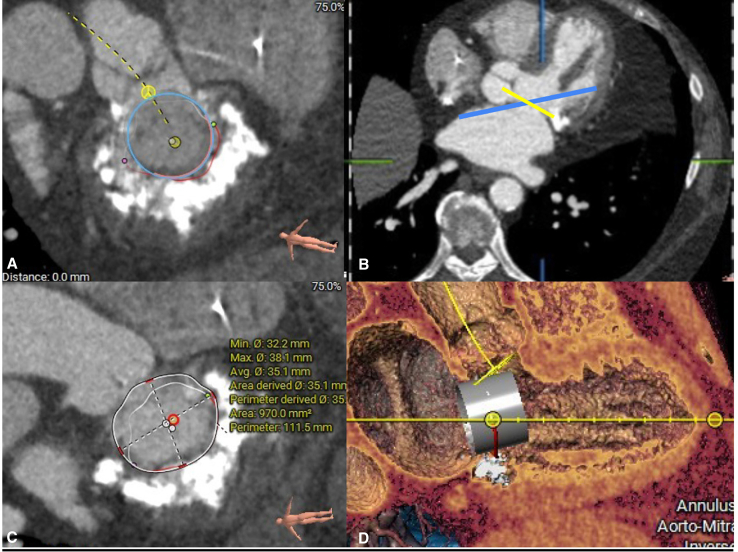
Computed tomography angiography (CTA) recon with mitral annular calcification (MAC) details and angle recon for case 1. A, Multiplanar reformatting (MPR) demonstrating severe MAC. The *yellow dotted line* represents deployment angle and distance. B, Axial multiplanar reformation MPR, mitral annulus (*yellow line*) with direction of nose cone on deployment system (*blue line*). C, MPR with planned landing zone (annulus area, 7.3 cm2; aorta-mitral angle, 63.4°; inverse angle, 116.6°). D, Volume rendering of double-oblique MPR demonstrating valve positioning. The *yellow dotted line* represents deployment angle and distance.

A 75-year-old woman with morbid obesity (body mass index, 41 kg/m^2^), diabetes, prior stroke, rheumatoid arthritis, pulmonary hypertension, complete heart block with a permanent pacemaker, and atrial fibrillation presented with worsening dyspnea. Structural heart computed tomography angiography (CTA) demonstrated severe MS with significant posterior MAC (Figure 1).

Operatively, there was severe MAC in the left atrium extending deep into the ventricle. The anterolateral muscle head was resected. A septal myectomy was performed to improve left ventricular outflow tract (LVOT) clearance. A #29 Sapien 3 Ultra Transcatheter valve was positioned, seated, and deployed at full volume (7 atm) using a soft J-wire through the Certitude delivery system. The valve was later postdilated over the wire with the deployment balloon at full volume. After weaning from CPB, the mean transmitral gradient was 1.8 mm Hg with trace paravalvular leak.

After weaning from CPB, the pericardium began filling with bright-red blood concerning for atrioventricular groove disruption or left ventricular (LV) injury. After recannulation and resumption of CPB, the patient was found to have an LV lateral wall injury with growing hematoma, attributed to a nose cone injury. A linear closure was performed using 2-0 Prolene horizontal mattresses with felt strips. A bovine pericardial patch was sewn to the LV wall, filled with Bioglue, and covered with a patch of Nu-Knit.

The patient was converted to central extracorporeal membrane oxygenation (ECMO) for LV decompression. ECMO was reconfigured to peripheral vessels on postoperative day (POD) 2 and decannulated on POD5. She was discharged after a prolonged postoperative course requiring eventual tracheostomy.

### Case 2

A 70-year-old woman with diabetes, end-stage renal disease, severe pulmonary hypertension, paroxysmal atrial fibrillation, heart failure with preserved ejection fraction, and calcific MS presented with shortness of breath. Structural CTA demonstrated severe MS with severe MAC encompassing the entire annulus and both leaflets, with a left ventricular ejection fraction of 55%, severe left ventricular hypertrophy, and torrential tricuspid regurgitation on transesophageal echocardiography (TEE).

Operatively, there was severe MAC throughout the annulus, with particularly severe calcium posteriorly. The valve leaflets were immobile and foreshortened, and the chordae were completely fused with the papillary muscles. After cutting the pericardium down inferiorly on the left to let the heart rest in the left chest, a #29 Sapien 3 Ultra transcatheter valve was introduced directly into the left atrium over a curved safari wire and deployed at 2 cc less than full volume (6 atm). Although there was no visible leak, there was concern given the difficult angulation of the delivery system in the chest. Examination revealed a possible tear at the LV apex anterolaterally with hematoma, so the valve was not postdilated (Video 1).

After closure of the atriotomy, the left ventricle was examined and patched with 2-0 Prolene sutures using 2 felt mattress strips, reinforced with a 4-0 Prolene, and then covered with a bovine pericardial patch sewn with a 4-0 Prolene and filled with Bioglue. The patient was then weaned from CPB. She was extubated on POD4. Her postoperative course was notable for acute-on-chronic kidney injury requiring chronic renal replacement therapy and complete heart block necessitating dual pacemaker implantation.

### Case 3

**Figure 2:**
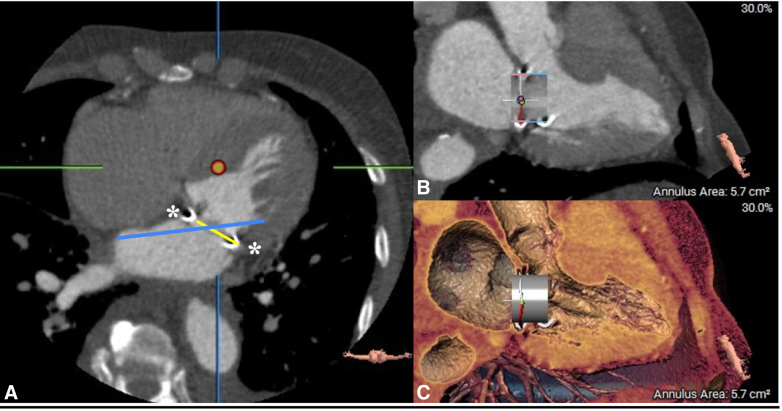
Computed tomography angiography (CTA) recon with mitral annular calcification (MAC) details for case 3. A, Axial multiplanar reformatting (MPR), mitral annulus (*yellow line*) with direction of nose cone on deployment system (*blue line*). Asterisks indicate prior mitral valve. B, Double-oblique MPR with reconstruction of valve positioning. C, Volume rendering of double-oblique MPR with reconstruction of valve positioning.

An 84-year-old woman with atrial fibrillation, chronic kidney disease, type 2 diabetes, a permanent thoracic catheter for chronic effusions, severe atheromatous disease with mobile atheroma at the arch of the aorta, and severe symptomatic mitral regurgitation from a prior tissue mitral valve replacement (#33 Hancock II placed 2 years earlier) complained of progressive dyspnea. She was at risk for LVOT obstruction (LVOT >300 mm^2^) and would require a leaflet modification procedure. Instead, she was evaluated for open surgical resection of mitral bioprosthetic leaflets and mitral valve-in-valve (Figure 2). TEE revealed severe mitral regurgitation, with an LVEF of 45% and anterior prolapse of the bioprosthetic mitral valve leaflet.

Operatively, the interatrial groove was opened, and the prior valve was found to have significant leaflet tears. The leaflets were resected, keeping the sewing ring in place. A Sapien XT 26 was prepared with crimping on a transapical Ascendra Plus delivery system with an extra 1 cc of volume added. Using CTA, the valve was sized as a #26 Sapien valve. Under direct vision, the delivery system was placed, and the valve was positioned across the valve frame without the use of a wire and deployed using slow balloon expansion with manual positioning adjustment. The Sapien was anchored well into the prior valve frame, with no paravalvular leak and good mitral valve function on TEE.

Shortly after arriving in the cardiothoracic intensive care unit, she developed severe bleeding through her mediastinal chest tubes. Reexploration revealed active bleeding from a perforation of the inferolateral wall of the left ventricle, likely due to the nose cone of the deployment system. CPB was reinstituted, and the LV perforation was closed with a linear closure using 2 Teflon felt strips and 2-0 monofilament sutures. TEE showed good function of the Sapien valve with no new regional wall motion abnormalities. The patient's postoperative course was unremarkable, and she was extubated on POD2 and discharged on POD9.

## Discussion

Hybrid transatrial transcatheter mitral valve replacement (TMVR) with direct valve implantation has been demonstrated to be safe and effective in patients with severe MAC in series^3^ and in the prospective single-arm SITRAL trial.^4^ Despite favorable short-term procedural success in high-risk patients,^5^ this approach should be reserved for patients who are not candidates for traditional mitral valve surgery.

In our institutional experience with transatrial TMVR, there were 65 patients (96%) without this injury. In these cases, the tissues were notably fragile and required extensive repair sutures for small tears. Other potential etiologies, such as guidewire perforation, have been reported with transcatheter aortic valve replacement, although these were unlikely in the present cases given the geometry of insertion.

Deployment of the balloon over a stiffer wire shaped on the back table to have a longer curve can deflect the nose cone apically, keeping the free wall relatively protected. The wire must be placed in the apex of the heart and not mid-ventricle, which can be challenging in a decompressed, thick heart. In deep chest spaces, lifting the left atrium through pericardial retraction sutures on the right ensures a direction of angulation of the balloon to the LV apex. We also divide the pericardium inferiorly on the left down to the phrenic nerve, which allows the LV apex to fall fully into the left chest. This is particularly important in a thick, hypertrophied ventricle to maintain a straight axis from mitral annulus to apex.

In both open scenarios of MAC and mitral valve-in-valve, a transcatheter valve is inserted through a surgical approach over a wire into the left ventricle. Having a nose cone in the ventricle in an arrested heart carries the potential for LV injury. This unique complication has not been described for fully transcatheter procedures, although LV injury related to inadvertent wire trauma or other factors has been reported.^6^ Challenges of managing a nose cone in a surgical approach are relaxing the heart to provide a straight trajectory from mitral annulus to apex and ensuring that the wire is at the LV apex. If these 2 criteria are not met, the risk of LV injury theoretically may increase. Furthermore, in case 3, the patient was noted to have a particularly deep chest cavity with a thick, hypertrophied ventricle and small left ventricle with a short distance from annulus to apex.

Other strategies to mitigate the risk of nose cone perforation include preferentially loading the transcatheter valve onto the distal portion of the delivery system balloon, which further shortens the length of the nose cone and reduces the risk of injury. We also have switched to exclusively using the transfemoral Commander system, which is more flexible compared to the more rigid Certitude system. Finally, in deep chests or unfavorable angles, a minimally invasive thoracotomy allows for excellent mitral exposure,^2^ with an optimal distance from annulus to the free wall. Repair should be undertaken on CPB with venting of the left ventricle. In our limited series, a horizontal mattress repair with felt, followed by patching of the left ventricle with a pericardial patch has worked well.

As novel approaches expand treatment options for high-risk patients, more TMVR recipients may require transatrial surgical access. LV nose cone perforation and subsequent major bleeding necessitating reintervention is not a widely reported complication. Successful outcome depends on early recognition and expedient surgical reintervention.

## Conflict of Interest Statement

Dr George serves as a consultant for Edwards Lifesciences and Medtronic and as a speaker for Boston Scientific. All other authors reported no conflicts of interest.

## Supplementary Data

Video 1 Operative video demonstrating valve deployment over soft J-wire in Case 1. Video available at: https://www.jtcvs.org/article/S2950-6050(25)00018-X/fulltext.
