# Supplementary figures and images for: Left ventricular perforation after transatrial delivery of a balloon-expandable valve
Source: JTCVS Struct Endovasc. 2025 Jul 3;7:100059. doi: 10.1016/j.xjse.2025.100059 (PMC13244730; doi:10.1016/j.xjse.2025.100059)

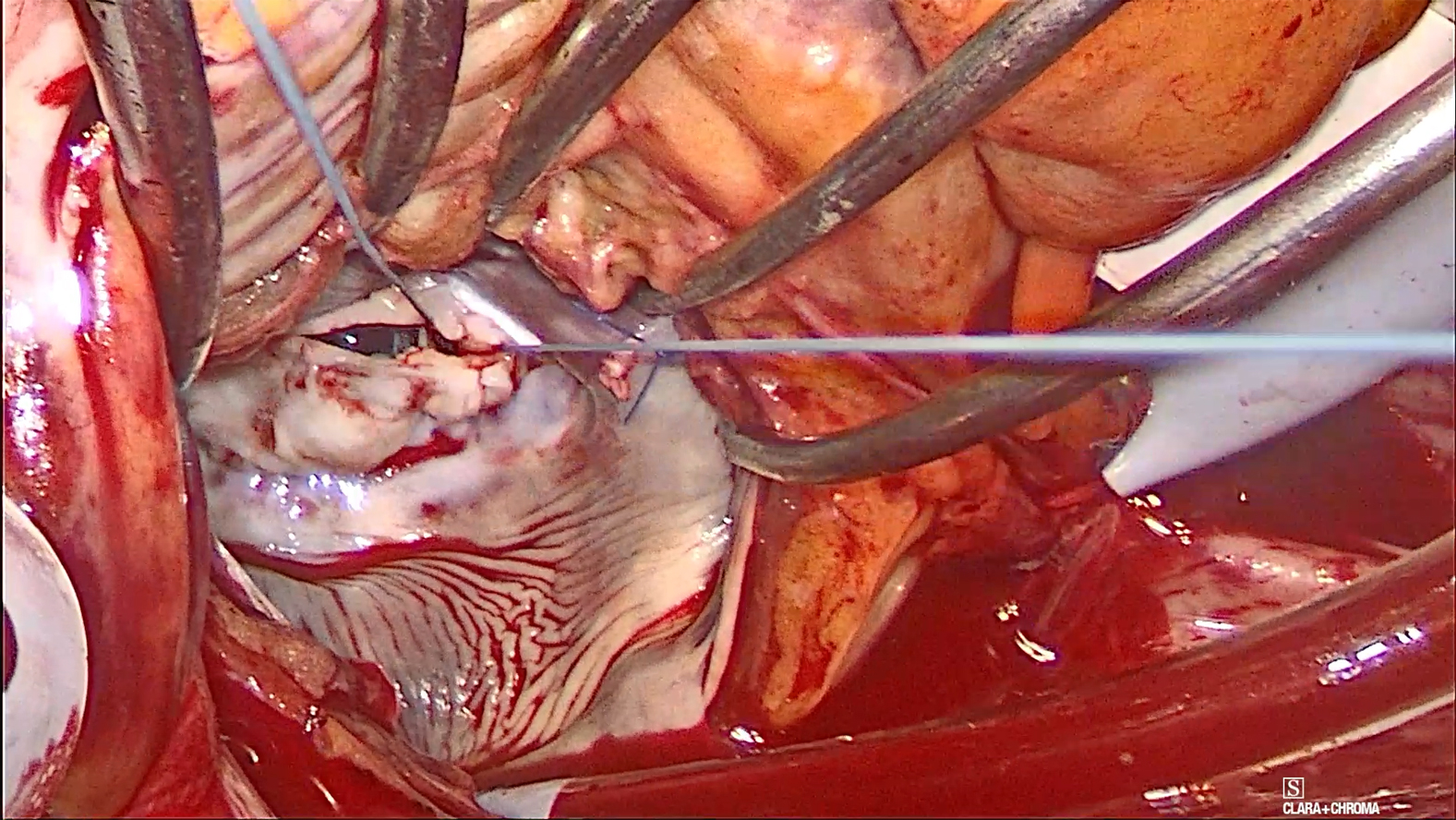

Supplement: Video 1 — Operative video demonstrating valve deployment over soft J-wire in Case 1. Video available at: https://www.jtcvs.org/article/S2950-6050(25)00018-X/fulltext. [file fx2.jpg]
